# Clinical outcome and influencing factors of differentiated thyroid cancer patients with radioiodine-refractory lung metastasis

**DOI:** 10.3389/fendo.2025.1622539

**Published:** 2025-08-04

**Authors:** Congcong Wang, Yutian Li, Guohua Qin, Jiao Li, Guoqiang Wang, Xinfeng Liu, Xufu Wang

**Affiliations:** ^1^ Department of Nuclear Medicine, The Affiliated Hospital of Qingdao University, Qingdao, Shandong, China; ^2^ Department of Radiology, Qingdao Women and Children’s Hospital, Qingdao, Shandong, China

**Keywords:** differentiated thyroid cancer, lung metastases, radioiodine therapy, radioactive iodine-refractory, prognosis

## Abstract

**Purpose:**

A subset of patients with differentiated thyroid cancer and lung metastases (DTC-LM) may progress to radioiodine-refractory (RAIR) disease, which is associated with a poor prognosis. This study aimed to investigate the clinical outcomes and potential risk factors associated with RAIR disease in DTC-LM patients.

**Methods:**

177 DTC-LM patients who underwent radioiodine (RAI) therapy at our center were retrospectively analyzed. Clinicopathological profiles were compared between the RAI-avid (RAIA) and RAIR groups. Univariate and multivariate regression analyses were conducted to identify risk factors for RAIR status and progressive disease (PD).

**Results:**

Overall, 80 patients were included in the RAIR group, accounting for 45.2% of the total patients. Multivariate analysis revealed that older age and higher T stage were independent risk factors for RAIR disease. Age≥55 years (HR: 2.975, 95% CI: 1.424 -6.218, P = 0.004), RAI-avid status (HR: 4.315, 95% CI: 1.753 - 10.622, P = 0.001) and the ps-Tg≥528.5ng/mL (HR: 3.665, 95% CI: 1.656 - 8.107, P = 0.001)were identified as independent predictors of PD. Kaplan–Meier analysis revealed a lower progression-free survival (PFS) rate in the RAIR group than in the RAIA group (P< 0.001).

**Conclusion:**

RAIR disease is common among DTC-LM patients and is associated with adverse clinical outcomes. Age, RAI avidity status, and ps-Tg levels serve as important predictors of PD. Early risk stratification and individualized management strategies are crucial to improving outcomes in DTC-LM patients.

## 1 Introduction

Thyroid cancer is one of the most common malignancies in adult patients, with an increased incidence rate in recent years (1, 2). According to 2015 national cancer epidemiology data analyzed by the National Cancer Center of China, the incidence of thyroid cancer reached 201,000 cases, with a population-adjusted rate of 14.6 per 100,000 (2). Differentiated thyroid cancer (DTC) accounts for over 95% of all thyroid cancer, and the majority of these cases have favorable prognoses, with 10-year survival rates exceeding 90% (3, 4).

Approximately 23% of DTC patients present with distant metastases either at diagnosis or during follow-up (5). Lung dissemination is the most common metastatic manifestation, accounting for 70% of distant metastasis cases (6, 7). Compared to non-metastatic counterparts, DTC patients with lung metastases (DTC-LM) exhibit significantly worse prognoses, with a median overall survival of less than 10 years (8, 9). In cases of DTC-LM with continuous radioiodine-avidity (RAI-avidity) at the metastatic site, RAI therapy remains one of the most effective treatment options (10, 11).

During the natural disease course or therapeutic interventions, one-third of DTC-LM patients exhibit tumor dedifferentiation, characterized by morphological and functional regression with loss of RAI-avidity, ultimately progressing to radioactive iodine-refractory (RAIR) disease (12–14). RAIR DTC-LM patients do not benefit from RAI therapy and show significantly poorer survival outcomes compared to their RAI-avid counterparts, with a median overall survival of 3–5 years and a 10-year survival rate of approximately 10% (8, 13). RAIR DTC-LM represents a critical therapeutic challenge in the management of DTC.

However, the limited research on RAIR disease has hindered a comprehensive understanding of its clinical implications. Therefore, the present study aims to systematically examine the clinical outcomes of DTC-LM patients with RAIR disease, as well as the relationships between clinicopathologic characteristics and RAIR disease. This investigation provides actionable insights for refining precision oncology frameworks in DTC-LM management.

## 2 Materials and methods

### 2.1 Patients

We retrospectively analyzed the medical records and clinical follow-up data of DTC-LM patients who underwent total or subtotal thyroidectomy combined with RAI from January 2000 to December 2021 at the nuclear medicine department of the Affiliated Hospital of Qingdao University. The inclusion and exclusion criteria for this study are detailed in **Table 1**. The study protocol was approved by the Affiliated Hospital of Qingdao University Ethics Committee and adhered to the principles of the Declaration of Helsinki, and the requirement for written informed consent was waived. All patient identifiers (e.g., names, hospital IDs) were removed at the point of data collection, and anonymized study IDs were used for analysis.

**Table 1:** Inclusion and Exclusion Criteria in this study.

Inclusion	Exclusion
Underwent total or subtotal thyroidectomy	Concomitant malignancy
Histopathological type was diagnosed as DTC	TgAb positive (TgAb ≥40 IU/mL)
Received at least one standardized RAI	Absence of critical data and follow-up information
LM and aged ≥18 years at the initial RAI	Receipt of other anti-tumor treatments (such as chemotherapy, external radiation therapy, targeted drug therapy, etc.).

DTC, differentiated thyroid carcinoma; RAI, radioiodine therapy; LM, lung metastasis; TgAb, anti-thyroglobulin antibody.

### 2.2 Data collection and RAI procedures

The clinical and pathological data included age, gender, histological type, multicentricity, primary tumor size, extrathyroidal extension (ETE), T stage, N stage, lymph node metastasis (LNM) ratio, post-operative stimulated thyroglobulin (ps-Tg), and the largest size of LM were collected. Then, each patient’s T and N stage were evaluated according to the 8th edition of the tumor-node-metastasis (TNM) classification by the American Joint Committee on Cancer (AJCC) (15, 16). All patients were confirmed to have LM based on chest CT findings, therapeutic ^131^I whole-body scan (Rx-WBS) results, thyroglobulin (Tg), and TgAb levels.

According to the 2015 American Thyroid Association (ATA) guidelines (10), all patients received one or more RAI therapies following total thyroidectomy. The dose of each RAI therapy ranged from 150–200 mCi, with appropriate adjustments based on the patient’s disease. Afterwards, Rx-WBS and single- photon emission computed tomography/computed tomography (SPECT/CT) were performed 3–7 days after RAI therapy. All patients were administered levothyroxine for TSH suppression after RAI therapy. Repeated RAI therapies was administered every 6 to 12 months, as long as the disease continued to concentrate RAI and showed clinical response.

According to the 2015 ATA guidelines (10), DTC-LM disease is categorized as either RAIR or RAI-avid (RAIA) disease. RAIR disease is classified under the following four conditions: i) DTC-LM lesions do not concentrate RAI on the first Rx-WBS, ii) DTC-LM lesions lose the ability to concentrate RAI after previously showing RAI avidity, iii) RAI is concentrated in some DTC-LM lesions but not others, and iv) DTC-LM lesions progress despite RAI uptake (12, 17).

### 2.3 Clinical outcome

**Figure 1:**
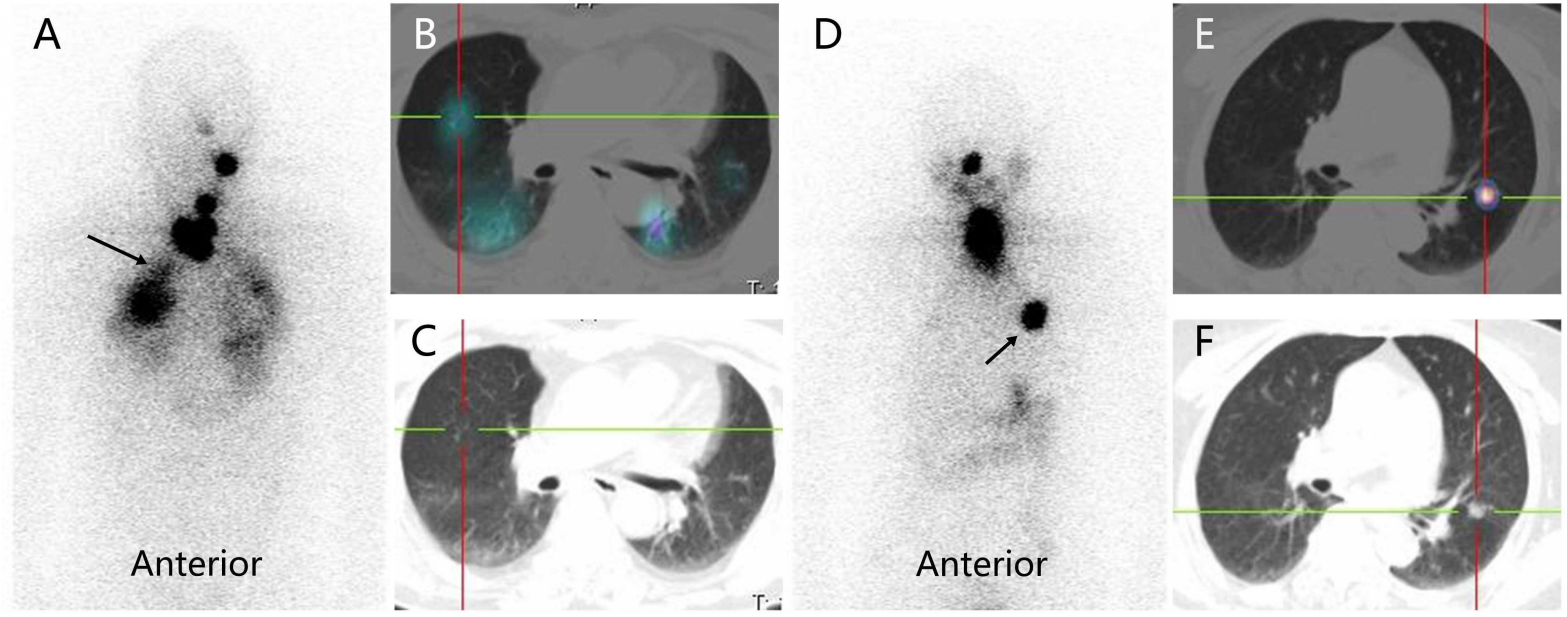
RAI-avid lung metastases from differentiated thyroid cancer in a male patient (RAI-avid DTC-LM lesions are visible only on Rx-WBS but not on chest CT) and a female patient (RAI-avid DTC-LM lesions are visible both on Rx-WBS and chest CT). **(A, D)** anterior Rx-WBS; **(B, E)** SPECT/CT fusion images post initial course of RAI therapy; **(C, F)** CT images post initial course of RAI therapy; RAI, radioiodine; DTC, differentiated thyroid cancer; LM, lung metastasis; Rx-WBS, therapeutic ^131^I whole-body scan.

The structural response of DTC-LM patients was assessed every six months to one year after RAI therapy using the Response Evaluation Criteria in Solid Tumors 1.1 (RECIST 1.1) (18). If RAI-avid DTC-LM lesions are visible only on Rx-WBS but not on chest CT (as shown in **Figure 1**), the patient will be excluded from the response assessment.

For DTC-LM patients with at least one measurable lesion (defined as LM lesions > 1 cm in diameter), the structural response was categorized into complete response (CR), partial response (PR), stable disease (SD), and progressive disease (PD) (**Table 2**). For patients with all LM lesions < 10 mm in diameter, the RECIST 1.1 criteria for non-target lesions were applied, classifying the response as CR, non-CR/non-PD, or PD (**Table 2**). Then, CR, PR, and SD (or CR and non-CR/non-PD) were grouped as non-progressive disease (NPD).

**Table 2:** The assessed structural response of DTC-LM patients in the response evaluation criteria in solid tumors 1.1 (RECIST 1.1).

Target lesions (at least one measurable LM lesion > 1 cm in diameter)	CR	the disappearance of all lesions
	PR	a ≥ 30% decrease in the sum of the largest diameter of all macronodular lesions compared to baseline, and no new lesions
	SD	lesions shrinkage or growth that does not meet the criteria for PR or PD
	PD	a ≥ 20% increase in the sum of the diameter of all macronodular lesions compared to baseline or the presence of one or more new lesions.
Non-target lesions (all existing LM lesions <10 mm in diameter)	CR	if all lesions disappeared and the suppressed serum thyroglobulin level was undetectable
	non-CR/non-PD	if one or more lesions existed persistently
	PD	if the number of lesions increased

LM, lung metastases; CR, complete response; PR, partial response; SD, stable disease; PD, progressive disease.

Progression-free survival (PFS) was defined as the time from the first detection of LM to either the determination of PD or the most recent assessment of therapeutic response in patients achieving NPD (17).

### 2.4 Statistical analysis

Statistical analyses were conducted using IBM SPSS software version 26.0. Data are presented as medians and interquartile ranges (IQR), minimum and maximum values, or frequencies and percentages. Binary logistic regression analysis was used to identify of independent predictors of RAIR. A Receiver-operating characteristic (ROC) curve was constructed to predict PD using ps-Tg, determining the optimal diagnostic threshold. Cox regression analysis was performed to identify independent predictors of PD. The PFS analysis was calculated from the date of the first detection of LM to either PD (RECIST 1.1 criteria) or last follow-up, with censoring applied for patients without events. Time-to-event data were analyzed using the Kaplan-Meier method, and between-group comparisons employed the log-rank test. All tests were two-tailed, and P< 0.05 was considered statistically significant.

## 3 Results

### 3.1 Baseline characteristics of DTC-lM patients

**Figure 2:**
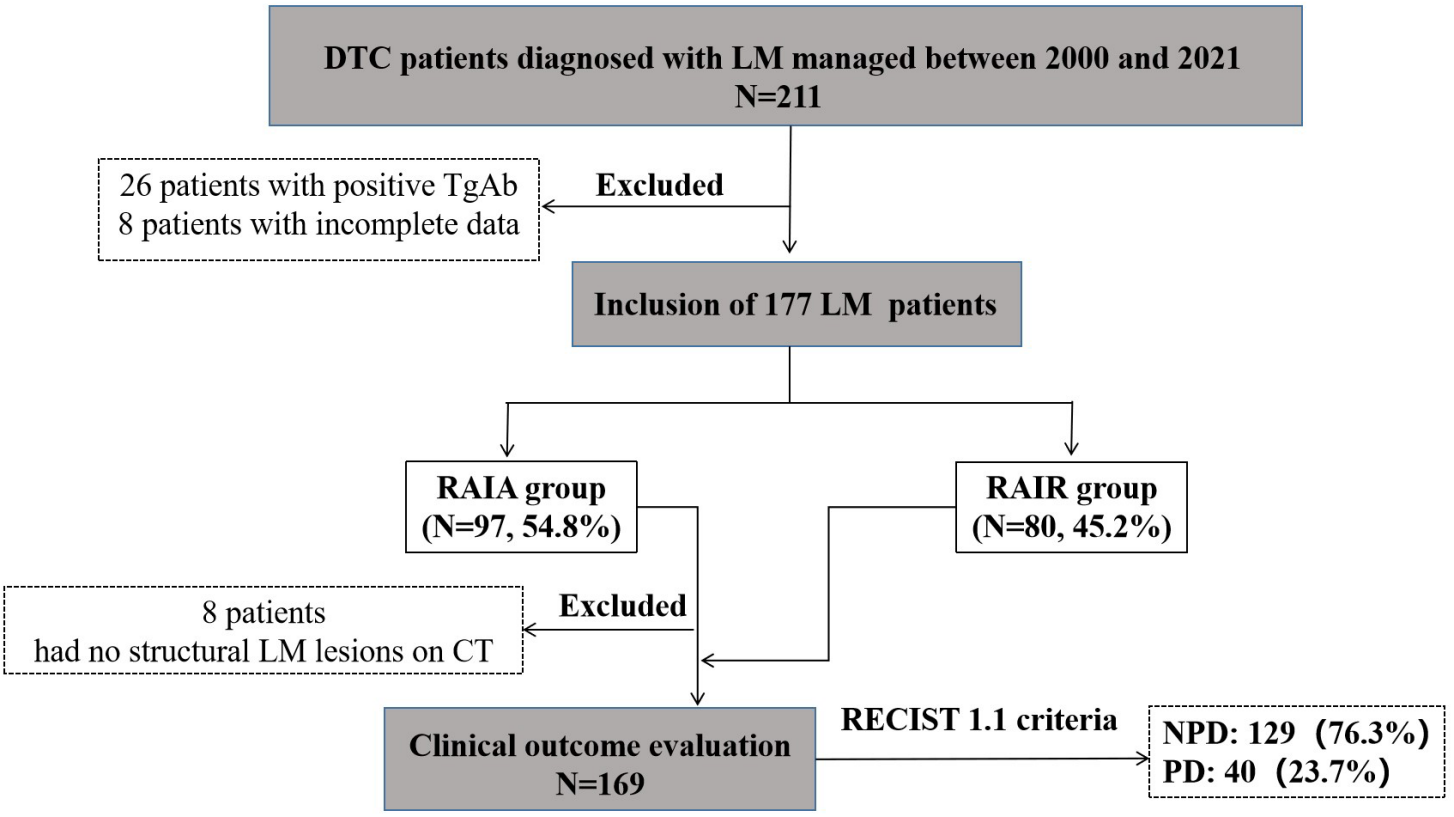
Selection flow chart and research roadmap of DTC-LM patients. DTC, differentiated thyroid cancer; LM, lung metastasis; RAIA, radioactive iodine avid; RAIR, radioactive iodine-refractory; PD, progressive disease; NPD, non-progressive disease.

From January 2000 to December 2021, 211 adult DTC patients were diagnosed with LM. After excluding 34 patients (26 patients with positive TgAb and 8 patients with incomplete data), the remaining 177 DTC-LM patients constituted our study cohort (**Figure 2**).

The baseline Clinicopathologic characteristics of 177 enrolled DTC-LM patients are summarized in **Table 3**. The median age of patients was 47 years (range: 18–76 years) and the majority were females (61.6%, 109/177). Among the cohort, 162 (91.5%) patients were diagnosed with papillary thyroid carcinoma (PTC), including 8 cases of follicular variant, 5 cases of tall cell variant, 4 cases of columnar cell variant, and 2 cases of diffuse sclerosing variant, while 15 (8.5%) patients were classified as follicular thyroid carcinoma (FTC). The size of the primary tumor ranged from 0.2 to 10.0 cm, with 13.6% (24/177) of patients having a larger size (≥4 cm). ETE was present in 55.4% (98/177) of patients. In addition, cervical lymph node metastases (LNM) occurred in 91.0% (161/177) of patients (N0-N1a=33; N1b =144).The LNM ratio ranged from 0% to 100%, with a median value of 38.1%. 53 (29.9%) patients with distant LM lesions > 1 cm in diameter. Regarding T staging, 71 patients (40.1%) were classified as T4. The ps-Tg levels ranged from 3.7 to 112,880.0 ng/mL, with a median of 109.8 ng/mL. One to six RAI therapy (median 2) were administered. The cumulative activity of RAI ranged from 150 mCi to 1050 mCi (median 350 mCi). The median duration of follow-up after the first RAI therapy was 74.4 months (range, 6.3-267.9 months).

**Table 3:** Clinicopathologic characteristics of DTC-LM patients (n=177).

Characteristics	Patients n (%)	Median (IQR)	Range
Age (years)		47 (36, 59)	18-76
<55	115 (65.0)		
≥55	62 (35.0)		
Gender			
Male	68 (38.4)		
Female	109 (61.6)		
Histological type			
PTC	162 (91.5)		
FTC	15 (8.5)		
Multicentricity			
Yes	98 (55.4)		
No	79 (44.6)		
Tumor size (cm)		2.0 (1.4, 3.0)	0.2-10.0
≤4cm	153 (86.4)		
>4cm	24 (13.6)		
ETE			
Negative	79 (44.6)		
Positive	98 (55.4)		
T stage			
T1/T2/T3	106 (59.9)		
T4	71 (40.1)		
N stage			
N0-N1a	33 (18.6)		
N1b	144 (81.4)		
Ratio of LNM		38.1% (20.8%, 57.8% )	0-100%
<50%	112 (63.3)		
≥50%	65 (36.7)		
Largest size of LM			
<1cm	124 (70.1)		
≥1cm	53 (29.9)		
ps-Tg (ng/mL)		109.8 (36.9, 500.0)	3.7-112880.0
Follow-up duration (months)		74.4 (42.0, 106.6)	6.3-267.9

LM, lung metastases; DTC, differentiated thyroid cancer; PTC, papillary thyroid cancer; FTC, follicular thyroid cancer; ETE, extrathyroidal extension; T, tumor; N, node; LNM, lymph node metastasis; ps-Tg, pre-ablation stimulated thyroglobulin.

### 3.2 RAIR disease in DTC-LM patients

According to the 2015 ATA guidelines, 80 patients were classified into the RAIR group, representing 45.2% of the total cohort. Among the RAIR patients, 42.5% (34/80) of these patients did not concentrate RAI at any point, followed by 27.5% (22/80) with partial RAI avidity, 21.2% (17/80) of patients who gradually lost their ability of RAI avidity, and 8.8% (7/80) of patients who experienced an increased number and size of LM despite RAI concentration.

### 3.3 Comparison of clinicopathological features between the RAIR and RAIA groups

**Table 4** compares the clinicopathological characteristics of the RAIA and RAIR groups. We found that patients aged ≥55 years (P<0.001), with ETE (P=0.019), T4 stage (P<0.001), higher ps-Tg levels (P=0.003), and the largest size of LM ≥1 cm (P=0.046) were more likely to have RAIR. However, no significant differences were observed between the RAIR and RAIA groups in other clinicopathological features, including gender, histological type, multicentricity, primary tumor size, N stage, and the ratio of LNM (all P > 0.05). Binary logistic regression analysis revealed that age ≥55 years and T4 stage were independent predictors of RAIR disease (**Table 4**).

**Table 4:** Univariate and multivariate analyses of predictors of RAIR in DTC-LM patients (n=177).

Characteristics	Univariate analysis				Multivariate analysis	
	RAIA (n=97)	RAIR (n=80)	*χ^2^/U*	*P*-value	OR (95% CI)	*P*-value
Age (years)			19.580	<0.001		
<55	77 (67.0%)	38 (33.0%)			Reference	
≥55	20 (32.3%)	42 (67.7%)			3.739 (1.891-7.393)	<0.001
Gender			0.154	0.694		
Male	36 (52.9%)	32 (47.1%)				
Female	61 (56.0%)	48 (44.0%)				
Histological type			0.438	0.508		
PTC	90 (55.6%)	72 (44.4%)				
FTC	7 (46.7%)	8 (53.3%)				
Multicentricity			0,155	0.694		
Yes	55 (56.1%)	43 (43.9%)				
No	42 (53.2%)	37 (46.8%)				
Tumor size (cm)			3.356	0.067		
>4cm	9 (37.5%)	15 (62.5%)			Reference	
≤4cm	88 (57.5%)	65 (42.5%)			0.411 (0.151-1.118)	0.082
ETE			5.481	0.019		
Negative	51 (64.6%)	28 (35.4%)			1.819 (0.640-5.170)	0.261
Positive	46 (46.9%)	52 (53.1%)			Reference	
T stage			15.824	<0.001		
T4	26 (36.6%)	45 (63.4%)			Reference	
T1/T2/T3	71 (67.0%)	35 (33.0%)			0.329 (0.170-0.636)	0.001
N stage			0.177	0.674		
N0-N1a	17 (51.5%)	16 (48.5%)				
N1b	80 (55.6%)	64 (44.4%)				
Ratio of LNM			0.258	0.611		
<50%	63 (56.2%)	49 (43.8%)				
≥50%	34 (52.3%)	31 (47.7%)				
Largest size of LM			3.974	0.046		
<1 cm	74 (59.7%)	50 (40.3%)			Reference	
≥1 cm	23 (43.4%)	30 (56.6%)			1.284 (0.617-2.672)	0.504
Ps-Tg	75.7 (28.4, 249.3)	173.2 (54.0, 1000.0)	2874.0	0.003		

RAIA, radioactive iodine avid; RAIR, radioactive iodine-refractory; PTC, papillary thyroid cancer; FTC, follicular thyroid cancer; ETE, extrathyroidal extension; T, tumor; N, node; LNM, lymph node metastasis; LM, lung metastases; ps-Tg, pre-ablation stimulated thyroglobulin.

### 3.4 Clinical outcomes

Among the 177 DTC-LM patients in the study cohort, 8 patients could not be evaluated for clinical outcome due to non-visible structural disease was observed on chest CT. The remaining 169 DTC-LM patients had assessable clinical outcomes (**Figure 2**). During follow-up with a median time of 75.8 months (range, 6.3-267.9 months), 40 (23.7%) patients showed progression to PD, 10 (5.9%) patients were classified as CR, and the remaining 119 patients (70.5%) were classified as non-CR/non-PD. Ultimately, 129 (76.3%) patients were grouped into the NPD group.

### 3.5 Predictive value of ps-Tg in PD at the last follow-up

**Figure 3:**
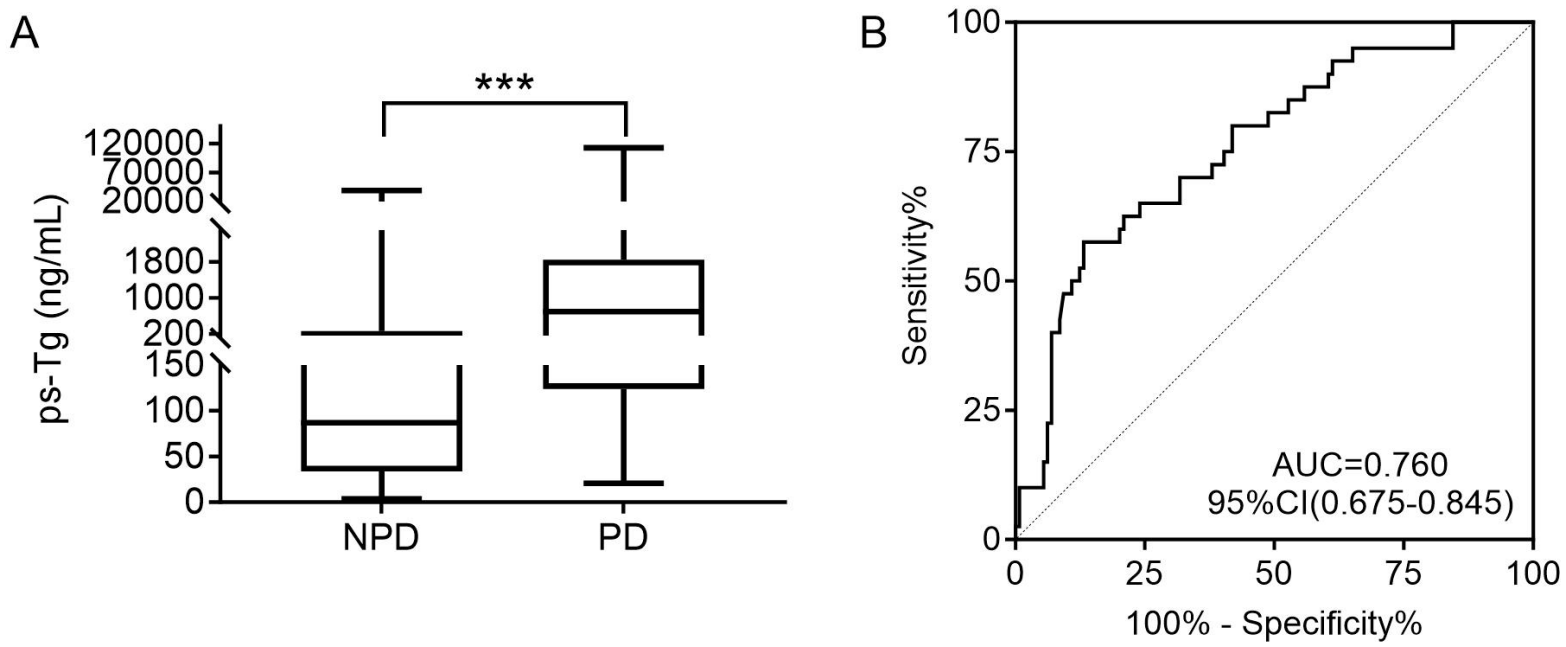
**(A)** Comparison of the ps-Tg level for PD and NPD patients. **(B)** ROC curves of ps-Tg for detecting PD. ps-Tg, pre-ablation stimulated thyroglobulin; PD, progressive disease; NPD, non-progressive disease; ROC, receiver operating characteristic; AUC, area under the ROC curve; ***P<0.001.

The ps-Tg level in the NPD group was 87.0 (33.6, 268.8) ng/mL, significantly lower than the 696.5 (123.8, 1846.0) ng/mL observed in the PD group (**Figure 3A**). Furthermore, ROC analysis identified a ps-Tg threshold of ≥528.5 ng/mL as the best threshold for discriminating PD from NPD at the last follow-up, with a sensitivity of 57.5%, specificity of 86.8%, and an AUC of 0.760 (95% CI: 0.675-0.845) (**Figure 3B**).

### 3.6 Comparison of clinicopathological features between the PD and NPD groups

**Table 5** compares the clinicopathological characteristics of the NPD and PD groups. Univariate analysis revealed that gender (P=0.028), age (P<0.001), primary tumor size (P=0.003), ETE (P=0.002), T stage (P<0.001), ps-Tg (P<0.001), RAI-avid status (P<0.001) and the largest size of LM (P<0.001) were associated with clinical outcomes. In multivariate analysis, the age≥55 years (HR: 2.975, 95% CI: 1.424 -6.218, P = 0.004), RAI-avid status (HR: 4.315, 95% CI: 1.753 - 10.622, P = 0.001) and the ps-Tg≥528.5ng/mL (HR: 3.665, 95% CI: 1.656 - 8.107, P = 0.001)were identified as independent predictors of PD (**Table 5**).

**Table 5:** Univariate and multivariate analyses of predictors of PD in DTC-LM patients (n=169).

Characteristics	Total (N)	Univariate analysis		Multivariate analysis	
		Hazard ratio (95% CI)	P value	Hazard ratio (95% CI)	P value
Gender					
Male	65	Reference		Reference	
Female	104	0.493 (0.262 - 0.928)	**0.028**	0.631 (0.310 - 1.284)	0.204
Age (years)					
<55	108	Reference		Reference	
≥55	61	3.234 (1.715 - 6.098)	**< 0.001**	2.975 (1.424 - 6.218)	**0.004**
Histological type					
PTC	154	Reference		Reference	
FTC	15	2.118 (0.933 - 4.811)	0.073	0.634 (0.219 - 1.835)	0.401
Multicentricity					
Yes	94	Reference			
No	75	1.135 (0.607 - 2.120)	0.692		
Tumor size (cm)					
≤4cm	147	Reference		Reference	
>4cm	22	2.886 (1.440 - 5.782)	**0.003**	1.651 (0.664 - 4.104)	0.280
Ratio of LNM					
<50%	107	Reference			
≥50%	62	0.872 (0.454 - 1.675)	0.681		
ETE					
Positive	93	Reference		Reference	
Negative	76	0.305 (0.145 - 0.642)	**0.002**	0.539 (0.156 - 1.865)	0.329
T stage					
T4	69	Reference		Reference	
T1/T2/T3	100	0.277 (0.143 - 0.537)	**< 0.001**	1.053 (0.333 - 3.334)	0.930
N stage					
N1b	139	Reference			
N0/N1a	30	0.877 (0.388 - 1.987)	0.754		
Ps-Tg					
<528.5ng/mL	129	Reference		Reference	
≥528.5ng/mL	40	5.660 (3.015 - 10.627)	**< 0.001**	3.665 (1.656 - 8.107)	**0.001**
RAI-avid status					
RAI-avid	97	Reference		Reference	
Partical or no RAI-avid	72	7.956 (3.511 - 18.030)	**< 0.001**	4.315 (1.753 - 10.622)	**0.001**
Largest size of LM					
<1 cm	116	Reference		Reference	
≥1 cm	53	2.913 (1.561 - 5.436)	**< 0.001**	1.279 (0.611 - 2.678)	0.515

LM, lung metastasis; PD, progressive disease; RAI, radioactive iodine; PTC, papillary thyroid cancer; FTC, follicular thyroid cancer; ETE, extrathyroidal extension; T, tumor; N, node; LNM, lymph node metastasis; LM, lung metastases; ps-Tg, pre-ablation stimulated thyroglobulin.

The bolded values indicate statistically significant results with P < 0.05.

**Figure 4:**
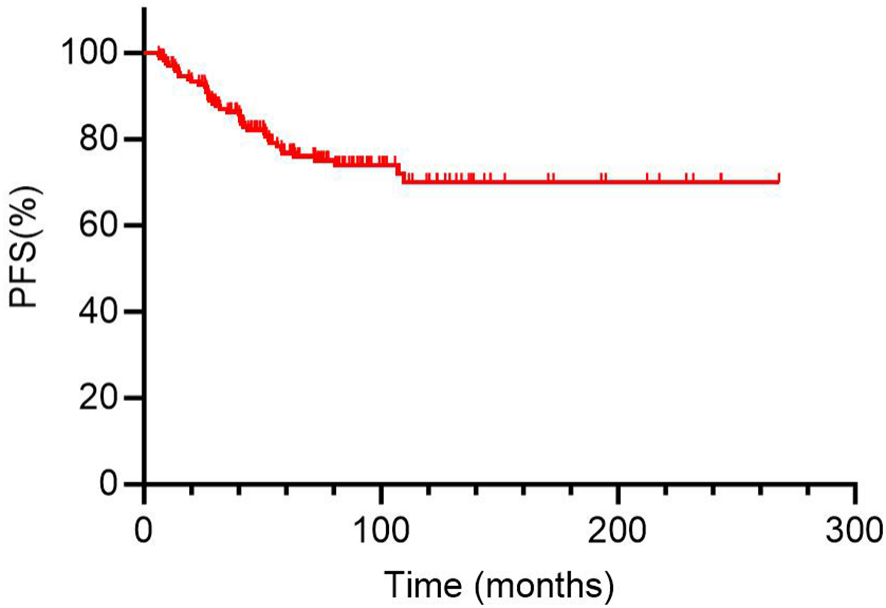
Kaplan-Meier curves for progression-free survival (PFS) of 169 DTC-LM patients. DTC, differentiated thyroid cancer; LM, lung metastasis.

**Figure 5:**
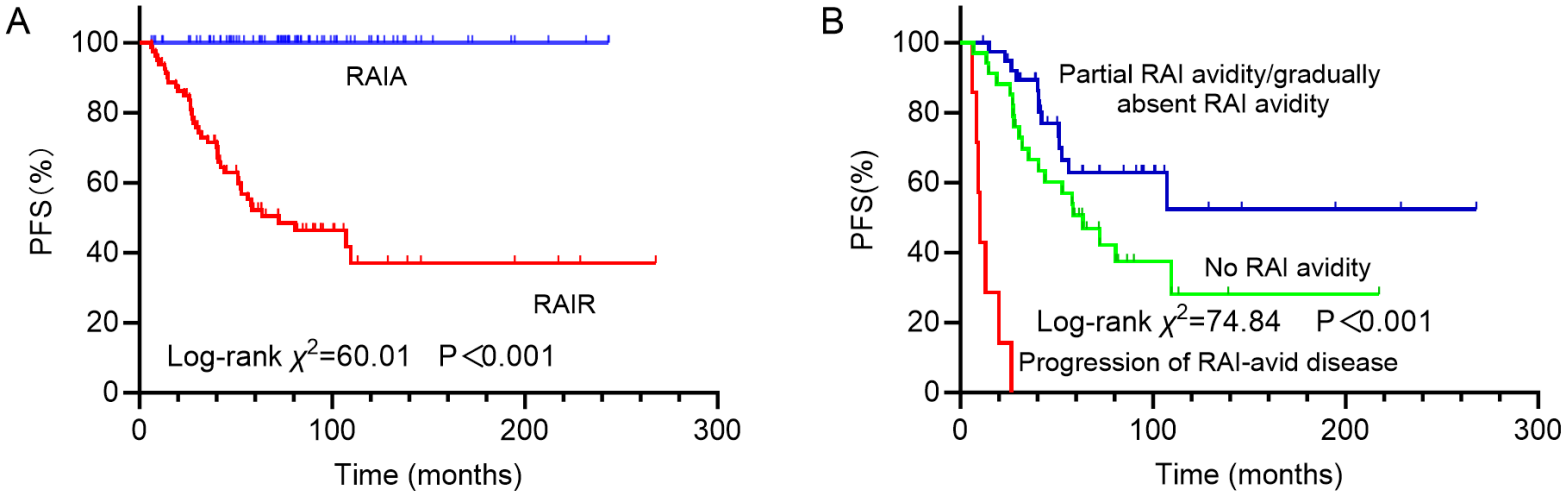
Kaplan-Meier curves for progression-free survival (PFS) of **(A)** RAIA and RAIR groups; **(B)** groups with no RAI avidity, partial RAI avidity/gradually absent RAI avidity and progression of RAI-avid disease. RAIA, radioactive iodine avid; RAIR, radioactive iodine-refractory; RAI, radioiodine.

In analysis of PFS, 40 patients had disease progression during follow-up. The median DFS was not reached. Then, the 1-year, 3-year, and 5-year PFS rates for the 169 DTC-LM patients were 97.0%, 86.3%, and 76.8%, respectively (**Figure 4**). As shown by the Kaplan-Meier curves, the 5-year PFS rates was 52.2% in the RAIR group and 100% in the RAIA group, with RAIR disease resulting in significantly poorer PFS (P < 0.001, **Figure 5A**).

Among the different subtypes of RAIR disease, PFS was poorest in patients with progressive disease despite LM lesions concentrating RAI, followed by those whose LM lesions never concentrated RAI, and was relatively more favorable in patients with partial RAI avidity or gradually absent RAI avidity, with 5-year PFS rates of 0%, 50.7% and 62.9%, respectively (P < 0.001, **Figure 5B**).

## 4 Discussion

LM is the most common distant metastasis in DTC. For DTC-LM patients, RAI therapy is recommended as the first-line choice unless the patient loses RAI accumulation capacity. Following RAI therapy, the 5-year survival rate for DTC-LM patients is 87.0%, and the 10-year survival rate is 69.2%. As a primary finding, we demonstrated that age ≥55 years and T4 stage are independent predictors of RAIR disease. Additionally, age ≥55 years (HR: 2.975, 95% CI: 1.424–6.218, P = 0.004), RAI-avid status (HR: 4.315, 95% CI: 1.753–10.622, P = 0.001), and ps-Tg ≥528.5 ng/mL (HR: 3.665, 95% CI: 1.656–8.107, P = 0.001) were identified as independent predictors of PD in DTC-LM patients.

In the present study, we identified age≥55 years as an independent risk factor for PD in DTC-LM patients. Sohn et al. (19) analyzed clinical data from 89 DTC patients with pulmonary metastasis and identified that age≥55 years as an independent risk factor for poor prognosis. Compared to patients aged <55 years, those aged ≥55 years had significantly lower PFS and cancer-specific survival rates. Additionally, Recent studies have commonly used 55 years as the cut-off age, a threshold also adopted by the AJCC/UICC TNM staging system to upstage patients (19–21). The consistent association between increasing age and worse prognosis in different studies highlights the importance of age as a key stratifying factor when assessing the treatment approach for DTC-LM patients (21).

Our study also found that a higher ps-Tg level (≥528.5 ng/mL) is an independent predictor of PD in DTC-LM patients. Serum Tg levels are widely used as a tumor marker in the risk assessment and management of DTC (10). The serum Tg level is associated with the extent of tumor burden. For every 1 g of neoplastic thyroid tissue, the Tg level rises by approximately 1 μg/L during thyroxine therapy, and by 2–10 μg/L following TSH stimulation (22, 23). Previous studies have established that rising Tg levels correlate with tumor progression, especially in patients with RAIR disease or metastasis (13). The 528.5 ng/mL ps-Tg threshold represents more than a statistical optimum, it reflects the tumor burden threshold where progression becomes likely despite ongoing RAI therapy (24). While assay-specific validation is recommended, this cutoff provides a clinically actionable benchmark for metastatic DTC management.

RAI therapy has been a cornerstone in the treatment of DTC. The presence of RAI avidity reflects a tumor’s capacity for iodine uptake, which is essential for the effectiveness of RAI therapy (19, 24, 25). Our study demonstrated that RAI-avid status, particularly the presence of partial or no RAI uptake, is an independent predictor of PD in DTC-LM patients. Tumors that show partial or no RAI uptake are often considered RAIR, which limits the efficacy of RAI therapy and is typically associated with worse clinical outcomes. Specifically, the 5-year PFS rate was markedly lower in the RAIR group (52.2%) than in the RAIA group (100%, P < 0.001), underscoring the adverse prognostic impact of RAIR status in DTC-LM patients. Further stratification of RAIR disease revealed substantial heterogeneity in PFS outcomes depending on the pattern of RAI avidity by LM. Notably, patients whose LM lesions retained RAI avidity but showed progressive disease had the worst prognosis, with a 5-year PFS rate of 0%. This may reflect a more aggressive tumor phenotype with higher metastatic potential despite preserved iodine uptake. In contrast, patients whose LM lesions never exhibited RAI avidity had an intermediate prognosis (5-year PFS: 50.7%), while those with partial or gradually decreasing RAI avidity showed relatively better outcomes (5-year PFS: 62.9%). These observations suggest that not all RAIR disease behaves uniformly, and that a nuanced classification based on RAI avidity patterns may provide additional prognostic information and guide therapeutic decision-making.

Partial RAI avidity in thyroid cancer lung metastases significantly impacts disease progression, representing an intermediate state with distinct biological behavior and clinical implications (10, 13). Compared to fully RAI-avid disease, partial RAI avidity carries a 3–4 fold increased risk of progression within 12 months (19). Patients with partial avidity may benefit from more frequent monitoring or earlier consideration of adjuvant therapies, as they represent a high-risk subgroup that may progress to RAIR disease (1).

Several previous studies have identified the size of LM—specifically, whether the diameter exceeds 1 cm, as an independent prognostic factor in DTC-LM patients (17, 19, 26). Larger metastatic lesions are generally associated with more aggressive tumor behavior, reduced RAI uptake, and poorer responses to standard radioiodine therapy, thereby correlating with unfavorable clinical outcomes. However, in our study cohort, LM lesion size did not emerge as an independent predictor of PD. This discrepancy may be attributed to differences in patient selection, sample size, or methodological approaches. Moreover, our findings underscore the dominant influence of RAI avidity, age and the ps-Tg levels on prognosis, which may override the prognostic significance of lesion size in certain clinical scenarios.

The study identified age≥ 55 years, higher T stage, and ps-Tg≥ 528.5 ng/mL as key predictors of RAIR disease, which can directly inform clinical practice by helping clinicians stratify DTC-LM patients into different risk categories during initial evaluation. These parameters should prompt more vigilant monitoring (e.g., shorter follow-up period) and earlier consideration of adjuvant therapies for high-risk patients, while potentially sparing low-risk patients from unnecessary aggressive treatments.

RAIR disease exhibits dedifferentiated characteristics with significant downregulation of thyroid-specific molecules, particularly the sodium-iodide symporter (NIS), whose reduced expression or functional impairment is most critical (13). RAIR disease typically develop from conventional DTC through a multi-step genetic evolution process. The transformation begins with early driver mutations in genes such as BRAF or RAS, or chromosomal rearrangements involving RET or NTRK3, followed by subsequent acquisition of secondary genetic alterations including TERT promoter mutations, TP53 mutations, PIK3CA mutations, and other late-stage molecular changes (13, 27). Importantly, specific genetic abnormalities, particularly TERT promoter mutations, PLEKHS1 promoter alterations, TP53 mutations, and 9q deletions have been strongly associated with the development of RAIR disease (28, 29).

For patients with symptomatic or rapidly progressive RAIR-DTC, multi-targeted kinase inhibitors (MKIs) are recommended as first-line therapy (10, 13). Prior to initiating targeted therapy, a comprehensive clinical evaluation of the patient should be conducted. In a prospective phase II study of RAIR-DTC, the combination of pembrolizumab (an anti-PD-1 monoclonal antibody) and lenvatinib achieved an objective response rate (ORR) of 62% and a 1-year PFS rate of 74% (30). However, whether this combination is superior to standard lenvatinib monotherapy requires validation through randomized controlled trials.

This study has several limitations. First, its retrospective design, combined with a relatively small study population derived from a single center, carries a potential risk of selection bias: the proportion of RAIR patients in our cohort was higher than that in population-based samples, and the exclusion of patients with incomplete follow-up data may have led to underrepresentation of those with different outcomes. Second, we did not include genetic profiling data (such as BRAF^V600E^, TERT, or RAS mutations), which could provide crucial insights into the molecular mechanisms underlying RAIR development. Third, while our data suggest clinically meaningful differences between RAIR subtypes, the small numbers in some subgroups preclude definitive conclusions. Fourth, our data were derived from a single tertiary medical center, which may affect the generalizability of our findings to broader populations. Future prospective, multicenter studies incorporating both clinical and molecular genetic data are warranted to validate and refine our findings.

## 5 Conclusion

In conclusion, RAIR disease was observed in approximately 45.2% of DTC-LM patients. Age and T stage were risk factors for RAIR disease. Furthermore, patients with partially or non–RAI-avid lesions, age ≥55 years, and ps-Tg levels ≥528.5 ng/mL tended to have poorer prognoses. These findings highlight the importance of early risk stratification and individualized management strategies based on clinical and biochemical parameters in DTC-LM patients.

## Data Availability

The original contributions presented in the study are included in the article/supplementary material. Further inquiries can be directed to the corresponding author.
